# Bacterial microbiome in the nose of healthy cats and in cats with nasal disease

**DOI:** 10.1371/journal.pone.0180299

**Published:** 2017-06-29

**Authors:** Elisabeth S. Dorn, Barbara Tress, Jan S. Suchodolski, Tariq Nisar, Prajesh Ravindran, Karin Weber, Katrin Hartmann, Bianka S. Schulz

**Affiliations:** 1 Clinic of Small Animal Medicine, LMU University of Munich, Munich, Germany; 2 Gastrointestinal Laboratory, Department of Small Animal Clinical Sciences, College of Veterinary Medicine and Biomedical Sciences, Texas A&M University, College Station, Texas, United States of America; Wageningen University, NETHERLANDS

## Abstract

**Background:**

Traditionally, changes in the microbial population of the nose have been assessed using conventional culture techniques. Sequencing of bacterial 16S rRNA genes demonstrated that the human nose is inhabited by a rich and diverse bacterial microbiome that cannot be detected using culture-based methods. The goal of this study was to describe the nasal microbiome of healthy cats, cats with nasal neoplasia, and cats with feline upper respiratory tract disease (FURTD).

**Methodology/Principal findings:**

DNA was extracted from nasal swabs of healthy cats (n = 28), cats with nasal neoplasia (n = 16), and cats with FURTD (n = 15), and 16S rRNA genes were sequenced. High species richness was observed in all samples. Rarefaction analysis revealed that healthy cats living indoors had greater species richness (observed species p = 0.042) and Shannon diversity (p = 0.003) compared with healthy cats living outdoors. Higher species richness (observed species p = 0.001) and Shannon diversity (p<0.001) were found in middle-aged cats in comparison to healthy cats in different age groups. Principal coordinate analysis revealed separate clustering based on similarities in bacterial molecular phylogenetic trees of 16S rRNA genes for indoor and outdoor cats. In all groups examined, the most abundant phyla identified were Proteobacteria, Firmicutes, and Bacteroidetes. At the genus level, 375 operational taxonomic units (OTUs) were identified. In healthy cats and cats with FURTD, *Moraxella* spp. was the most common genus, while it was unclassified *Bradyrhizobiaceae* in cats with nasal neoplasia. High individual variability was observed.

**Conclusion:**

This study demonstrates that the nose of cats is inhabited by much more variable and diverse microbial communities than previously shown. Future research in this field might help to develop new diagnostic tools to easily identify nasal microbial changes, relate them to certain disease processes, and help clinicians in the decision process of antibiotic selection for individual patients.

## Introduction

Microorganisms, including bacteria, fungi, and viruses, colonize the entire body. To understand their complex community structure, biology, and ecology, analyses of the microbial diversity of the body are important [1]. The microbiome is defined as the collection of microbes and their genomes, such as bacteria, archaea, viruses, and fungi, which can be either symbiotic, pathogenic, or commensal [2]. In humans, a subject’s microbiome is personalized but dynamic throughout the first year of life [3]. The microbiome is a metabolically active organ with the potential to influence both the physiology and phenotype of the host [4]. Most interactions between humans and their microorganisms are not disease-related [5], and instead most microorganisms live in a symbiotic relationship with their host [6]. It is known that the microbiome supports the stimulation, development, and modulation of the immune system [7]. Furthermore, it influences the structure of the mucosa and skin and prevents its host from being colonized by potentially pathogenic microorganisms [8]. However, an imbalance of the microbiome can result in damage to its host [9]. In the last decade, a number of studies have reported compositional alterations in the microbiome of the nose of healthy and diseased humans [10–28]. The development [29–31] and influence of environmental factors [32, 33] on the nasal microbiome during childhood and changes in the nasal microbiome during aging [34, 35] have also been the subject of different studies.

Changes in the microbial populations in the nose of animals have traditionally been evaluated using conventional microbiological techniques such as culture and biochemical methods [36]. Recent molecular-based methods, most commonly targeting the 16S rRNA gene, have enabled researchers to characterize highly complex microbial communities in different sites of the human body [37–39]. In small animal medicine, most of the our knowledge about the microbiome is based on analyses of 16S rRNAs from the gastrointestinal tract [40]. In contrast, very little information is available for the respiratory tracts of dogs [41] and cats [42]. The upper and lower airways are in permanent contact with the external environment during respiration and are therefore considered to be inhabited by mucosal commensals in healthy animals [43].

This study was designed to examine the microbiomes of healthy cats, cats with nasal neoplasia, and cats with feline upper respiratory tract disease (FURTD) using next-generation sequencing techniques. Furthermore, the influence of different individuals or environmental factors on the feline nasal microbiome of healthy cats was evaluated.

## Materials and methods

### Study population and inclusion criteria

This study was approved by the ethics committee of the Center for Clinical Veterinary Medicine, Faculty of Veterinary Medicine, LMU Munich, and has been assigned number 25-30-04-2014. Healthy cats (n = 28), cats with nasal neoplasia (n = 16), and cats with FURTD (n = 15) were included in the study. All samples were obtained from the federal state of Bavaria, southern Germany, between November 2014 and September 2015. Information regarding breed, age, sex, vaccination status, inside/outside status, duration of clinical signs, additional diseases, and current therapy were documented. A general physical examination and specific examination of the respiratory tract were performed in each cat. Patients were divided into different age groups (group 1: 0–0.3 years, group 2: >0.3–1.0 years, group 3: >1.0–5.0 years, group 4: >5.0–10.0 years, group 5: >10.0 years).

Healthy cats had to be clinically healthy for 6 months prior to sampling and had not been treated with antibiotics, anti-inflammatory, or immunosuppressive drugs during the last 6 months according to the exclusion criteria from the NIH Human Microbiome Project [44]. Healthy cats younger than 1 year were only included for comparison of age-related statistics because different human studies [3] and a longitudinal study on pigs [43] showed significant dynamic changes in the nasal microbiome in early life. Furthermore, a longitudinal study examining the fecal microbiome of cats younger than 1 year revealed higher structural and functional diversity of the microbiome later in life [45].

Cats with nasal neoplasia were eligible to enter the study if the histopathology of nasal biopsy samples confirmed malignancy. Cats with a history of antibiotic pretreatment were only included for comparison to pretreated and untreated animals because human studies showed alterations of the microbiome in individuals who received antibiotic treatment [46–48].

Cats with FURTD were eligible to enter the study if they had at least one clinical sign associated with FURTD, including nasal discharge, sneezing, ulceration of the tongue, conjunctivitis, ocular discharge, keratitis, and corneal ulcers. Only cats with an acute history of FURTD of less than 4 weeks were included. Furthermore, the cats had to test positive for at least 1 pathogen associated with FURTD, including feline herpesvirus-1 (FHV-1), feline calicivirus (FCV), or *Chlamydia felis* (*C*. *felis*). The cats had not been treated with antibiotic, anti-inflammatory, or immunosuppressive drugs during the prior 6 months. None of the cats involved in the study had received intranasal vaccines, which were an exclusion criterion.

#### Patient population

The ages of the healthy cats (Table 1) ranged from 6.0 months to 14.0 years (median 4.0 years). The median weight was 3.5 kg (1.3 kg to 7.5 kg). Healthy cats were either client-owned (n = 19), from animal shelters (n = 6) or farm cats (n = 3).

**Table 1:** Characteristics and environmental factors for the healthy cats enrolled in the study.

population	breed	age (years)	age group	sex	anesthesia	indoor/ outdoor	environment	relation	house
cat1	DSH	4.0	3	SF	W	I	I	-	-
cat2	DSH	3.0	3	CM	W	I	I+B	-	1
cat3	R-Mix	2.0	3	SF	W	I	I+B	-	1
cat4	DSH	5.7	4	SF	W	O	O	1	2
cat5	DSH	6.6	4	F	W	O	O	1	2
cat6	DSH	4.7	4	CM	W	O	O	1	2
cat7	DSH	2.6	3	F	W	O	O	1	2
cat8	DSH	10.0	5	SF	W	O	O	-	-
cat9	BSH-Mix	5.0	4	CM	W	I	I	2	3
cat10	BSH-Mix	5.0	4	SF	W	I	I	2	3
cat11	BE	4.0	3	SF	W	I	I+B	-	-
cat12	DSH	5.7	4	CM	W	O	O	1	4
cat13	DSH	14.0	5	SF	W	O	O	-	4
cat14	DSH	0.7	2	M	A	O	F	3	5
cat15	DSH	0.7	2	F	A	O	F	3	5
cat16	DSH	7.0	4	SF	W	O	O	4	6
cat17	DSH	7.0	4	CM	W	O	O	4	6
cat18	DSH	6.0	4	SF	W	O	O	-	-
cat19	DSH	2.0	3	CM	W	I	I+B	5	7
cat20	DSH	2.0	3	CM	W	I	I+B	5	7
cat21	DSH	1.0	3	F	A	I	S	-	8
cat22	DSH	8.0	4	SF	A	I	S	-	-
cat23	DSH	1.5	3	F	A	I	S	-	9
cat24	DSH	2.8	3	F	A	I	S	-	9
cat25	OSH	6.0	4	SF	A	I	S	-	8
cat26	DSH	1.5	3	F	A	I	S	-	8
cat27	DSH	0.5	2	M	W	O	F	-	-
cat28	DSH	3.0	3	CM	W	I	I	-	-

DSH: Domestic Shorthair, R-Mix: Ragdoll mix, BSH-Mix: British Shorthair mix, BE: Bengal; OSH: Oriental Shorthair; age groups: group 1: 0–0.3 years, group 2: >0.3–1 year, group 3: >1–5 years, group 4: >5–10 years, group 5: >10 years; M: male, CM: male castrated, F: female, SF: female spayed; W: sampled without anesthesia, A: sampled during anesthesia; I: indoor, O: outdoor, I+B: indoor with access to a balcony (indoor), S: shelter (indoor), F: farm (outdoor); relation: related cats were assigned the same number; house: cats housed together were assigned the same number.

The age of the cats with nasal neoplasia (Table 2) ranged from 3.5 years to 20.5 years (median 10.4 years), and the median weight was 4.1 kg (2.2 kg to 7.8 kg). All cats were sampled during anesthesia for diagnostic work-up at the Clinic of Small Animal Medicine of the LMU University of Munich.

**Table 2:** Characteristics and pretreatment of cats with nasal neoplasia enrolled in the study.

population	breed	age (years)	age group	sex	histopathology	antibiotics	steroids
cat29	B	10.0	5	CM	L	Y	Y
cat30	L	12.0	5	F	SCC	N	N
cat31	DSH	12.0	5	CM	L	Y	Y
cat32	DSH	8.0	4	CM	L	Y	Y/T
cat33	DSH	7.0	4	CM	L	N	Y/T
cat34	DSH	14.0	5	CM	F	N	N
cat35	DSH	9.0	4	SF	L	Y/T	Y/T
cat36	DSH	17.0	5	CM	L	Y	N
cat37	MC	5.0	4	CM	SCC	N	Y
cat38	DSH	3.5	3	F	SCC	N	N
cat39	B	10.7	5	F	AD	Y/T	Y
cat40	DSH	7.6	4	F	SCC	Y	N
cat41	DSH	20.5	5	SF	C	N	N
cat42	DSH	12.2	5	SF	SCC	Y	Y
cat43	DSH	12.7	5	CM	AD	Y/T	N
cat44	DSH	8.3	4	CM	L	N	N

DSH: Domestic Shorthair, B: Birman, L: Domestic Longhair, MC: Maine Coon; age groups: group 1: 0–0.3 years, group 2: >0.3–1 year, group 3: >1–5 years, group 4: >5–10 years, group 5: >10 years; M: male, CM: male castrated, F: female, SF: female spayed; N: no treatment for at least 3 months prior sampling, Y: yes, Y/T: yes and currently under treatment; SCC: squamous cell carcinoma, L: lymphoma, F: fibrosarcoma, C: carcinoma, AD: adenocarcinoma.

The age of the cats with FURTD (Table 3) ranged from 1.0 month to 6.6 years (median 0.3 years), and their median weight was 1.2 kg (0.3 kg to 4.0 kg). All cats were client-owned and sampled without anesthesia.

**Table 3:** Characteristics and infection status of cats with FURTD enrolled in the study.

population	breed	age (years)	age group	sex	pathogens
cat45	T	1.0	3	F	FHV
cat46	DSH	4.0	3	SF	FHV
cat47	DSH	6.6	4	F	FCV
cat48	DSH	0.1	1	M	FCV
cat49	DSH	0.2	1	M	FCV, *C*. *felis*
cat50	DSH	0.2	1	M	FHV
cat51	DSH	0.3	2	F	FCV
cat55	DSH	0.2	1	M	*C*. *felis*
cat56	DSH	0.2	1	F	*C*. *felis*
cat57	DSH	0.3	2	F	*C*. *felis*
cat58	DSH	0.2	1	M	*C*. *felis*
cat59	DSH	0.2	1	F	*C*. *felis*
cat60	DSH	0.4	2	F	*C*. *felis*
cat61	DSH	0.4	2	F	*C*. *felis*
cat62	DSH	0.5	2	M	FCV

DSH: Domestic Shorthair, T: Toyger; age groups: group 1: 0–0.3 years, group 2: >0.3–1 year, group 3: >1–5 years, group 4: >5–10 years, group 5: >10 years; M: male, CM: male castrated, F: female, SF: female spayed.

### Sampling technique

Two dry sterile swabs consisting of short nylon fibers (Copan^®^, FLOQSwabs^TM^, Brescia, Italy) were collected per cat, 1 from each nostril. The samples were obtained by gently inserting and rotating the swab into the cranial aspects of the nasal cavity. Samples were stored at -80°C until analyses were performed. One additional sterile dry rayon swab (Copan^®^ sterile dry swab 155C, Brescia, Italy) was obtained from the cats with FURTD from the conjunctiva, nares, pharynx, and tongue, and submitted for viral testing (IDEXX laboratories, Ludwigsburg, Germany).

### DNA isolation

Total nucleic acid (DNA) isolation using the QIAamp^®^ DNA Mini Kit (Qiagen, Hilden, Germany) was performed according to the manufacturer’s instructions and as described previously [49]. Samples from the left and right nostril of each cat were pooled. The swabs were placed in a 2-ml tube filled with phosphate-buffered saline (PBS) supplemented with 0.1% NaN_3_ as a fungicide. The samples were incubated at room temperature for 3 hours. The swabs were removed from the tubes, and the remaining buffer solution was centrifuged (Eppendorf Centrifuge 5417R, Hamburg, Germany) for 10 minutes at 7,500 rpm. After centrifugation, the supernatant was removed, and the pellet was resuspended in 180 μl of buffer ATL. After addition of 20 μl proteinase K, the sample was mixed by vortexing and incubated in a 1.5-ml reaction tube at 56°C (Eppendorf Thermomixer comfort, Hamburg, Germany) for 1 hour. The tube was briefly centrifuged at 7,500 rpm, followed by the addition of 200 μl buffer AL and vortexing. The tube was incubated at 70°C for 10 minutes and briefly centrifuged. Afterwards, 200 μl ethanol was added, and the tube was vortexed and briefly centrifuged. The mixture was applied to the QIAamp Mini spin column and centrifuged at 800 rpm for 1 minute. Samples from both nostrils of each cat were pooled, placed on the same QIAamp Mini spin column and briefly centrifuged. The QIAamp Mini spin was placed in a sterile 2-ml collection tube and centrifuged briefly. DNA was washed by adding 500 μl Buffer AW1, centrifuging at 8,000 rpm for one minute, and transferring the sample to a new 2-ml collection tube. Next, 500 μl buffer AW2 was added, and the tube was centrifuged at 14,000 rpm for 3 minutes. The QIAamp Mini spin column was placed in a new 1.5-ml microcentrifuge tube followed by the addition of 100 μl buffer AE, and the sample was incubated at room temperature for 5 minutes. The QIAamp Mini spin column was centrifuged at 8,000 rpm for 1 minute. As a negative control, the same procedure was performed using sterile water with and without an unused swab. The extracted DNA was stored at—80°C until further analysis.

### DNA sequencing

Sequencing based upon the V4 region of the 16S rRNA gene was performed on an Illumina MiSeq instrument (Illumina Inc.; San Diego CA, USA) at the MR DNA Laboratory (Shallowater, TX, USA) as described previously, with forward primer 515 and reverse primer 806 [50, 51]. Raw sequence data were screened, trimmed, filtered, denoised, and chimera-depleted with default settings using QIIME pipeline version 1.8 software (http://qiime.sourceforge.net) [52] and UCHIME (http://www.drive5.com/uchime) [53]. Operational taxonomic units were defined as bacterial sequences with at least 97% similarity to representative sequences from the Greengenes v 13.8 database [54]. Sequences were clustered into OTUs by using an open reference approach in QIIME [54]. All sequences from all samples were clustered into operational taxonomic units (OTUs), which is based on sequence similarity within the reads. Accordingly, OTUs in QIIME are clusters of sequences that represent some degree of taxonomic relatedness. For example, when sequences are clustered at 97% sequence similarity, each resulting cluster is typically thought of as representing a genus. These current techniques for selecting OTUs are known not to concur with the original term “species.”. In this context, the “observed species” metric is the number of unique OTUs found in the samples [52]. Data were uploaded to the database of the National Center for Biotechnology Information (NCBI) (accession number SRP074617).

### Data analysis

A total of 4,760,303 sequences (median 73,763; mean 74,380; range 25,641–126,171) were amplified. All samples were rarefied to an equal depth of 25,640 sequences per sample. Data were used to define the relative percentages of bacteria for each individual sample. The alpha and beta diversity were measured, and principal coordinates analysis (PCoA) plots and rarefaction curves were created using QIIME v1.8 software (Knight and Caporaso Labs, Arizona, USA). Weighted and unweighted UniFrac analyses were performed. Differences in microbial communities between the groups were investigated by analysis of similarity (ANOSIM) calculated for unweighted and weighted UniFrac distances using the PRIMER 6 statistical software package (PRIMER-E Ltd., Luton, UK). P-values <0.05 were considered statistically significant.

Assumption of normality was tested using the D’Agostino and Pearson normality test (Prism v.7.0, Graph-Pad Software Inc., CA, USA). As most datasets did not meet the assumptions of a normal distribution, differences in the proportions of bacterial taxa (defined as percentage of total sequences) between groups were determined using the nonparametric Kruskal–Wallis test or for comparison of only 2 groups the Mann-Whitney test (Prism v7.0, Graph-Pad Software Inc., CA, USA). The resulting p-values were adjusted for multiple comparisons using the Benjamini Hochberg’s false discovery rate (FDR). Dunn’s multiple comparison test was used to determine which of these groups were significantly different when age groups and environment was compared. The phylogeny-based UniFrac distance metric analysis was used to investigate differences in microbial communities between groups [54]. Linear discriminant analysis (LDA) effect size (LEfSe) was utilized to evaluate differentially abundant bacterial taxa and predicted function among the animal groups. This analysis was performed online (https://huttenhower.sph.harvard.edu/galaxy/).

## Results

### Study population

Healthy cats younger than 1 year (n = 3) were excluded for all investigations other than those that examined the effect of age on the microbiome. When excluding healthy cats younger than 1 year, the age of healthy adult indoor (median 3.0 years) and outdoor cats (median 5.7 years) was significantly different (p = 0.002). Another significant difference (p<0.001) was observed when comparing the age of healthy cats (median 4.0 years) and cats with FURTD (median 0.3 years), and those with nasal neoplasia that did not receive antibiotics (median 8.3 years) and cats with FURTD. However, there were no significant differences regarding age when healthy cats and cats with nasal neoplasia were compared according to Dunn’s multiple comparison test.

### Nasal microbial composition

**Fig 1:**
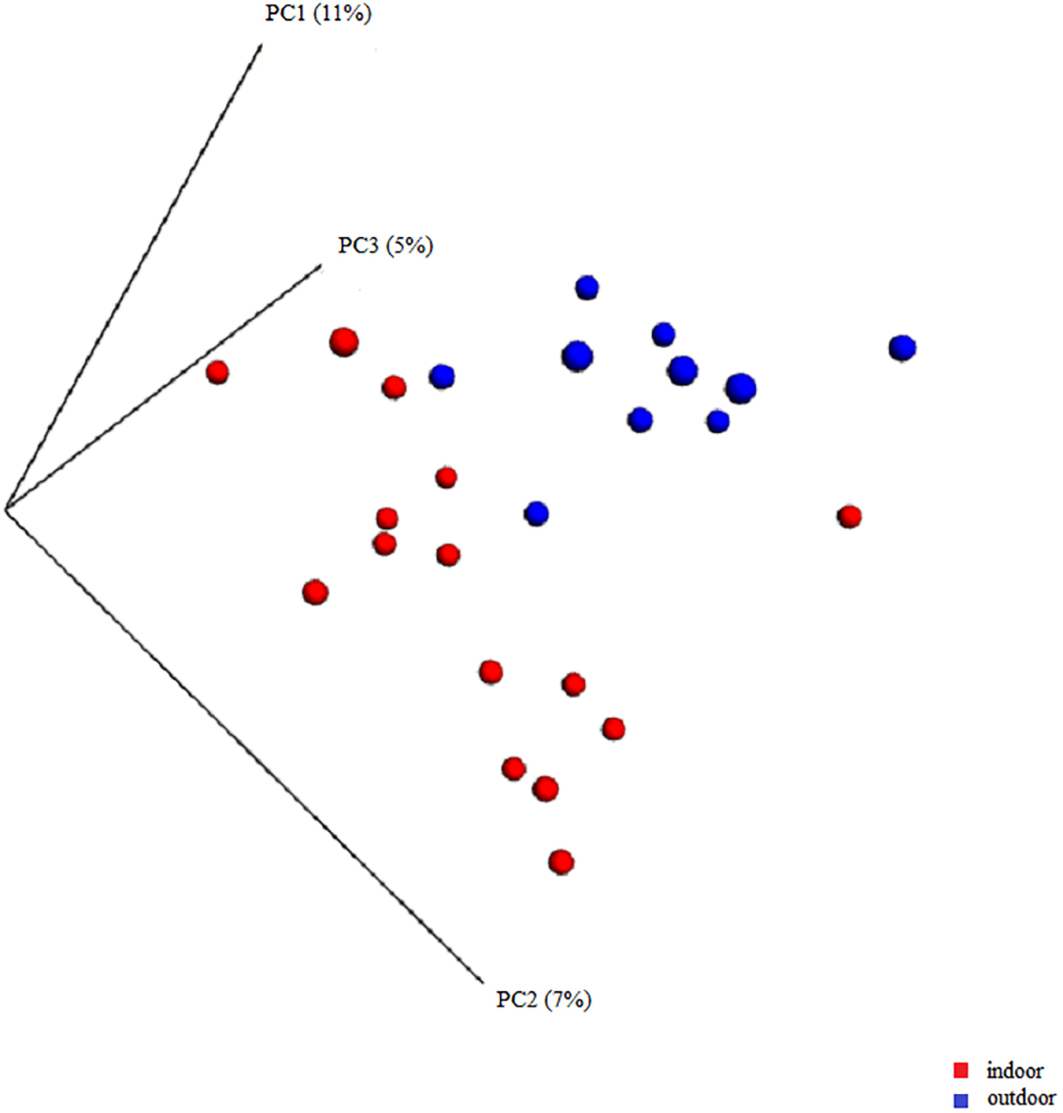
Principal coordinate analysis for indoor/outdoor status of healthy cats. Principal coordinate analysis (PCoA) plots based on the unweighted UniFrac distance metric illustrating differences in microbial communities present in the nose of healthy cats living indoor versus outdoor. Every dot represents the bacterial community of one individual cat. A separation defined as clustering was observed in indoor and outdoor cats. PC1: first component along the x- and y-axes, PC2: second component, PC3: third component; blue dots: outdoor cats; red dots: indoor cats.

The following factors were considered in the analysis of nasal microbial composition of healthy cats: the influence of age (age groups 1–5), environment (indoor/outdoor), group housing, familial relationship between cats, and sampling with or without anesthesia. When all types of environments, such as indoor, indoor and balcony, shelter, and outdoor (ANOSIM on unweighted UniFrac distance p = 0.003 and on weighted UniFrac distance p = 0.001), just indoor and outdoor (ANOSIM on unweighted UniFrac distance p = 0.001 and on weighted UniFrac distance p = 0.015) and different age groups (ANOSIM on unweighted UniFrac distance p = 0.002 and on weighted UniFrac distance p = 0.048) were compared, these groups were significantly different. Pairwise testing using PRIMER6 indicated that cats living indoors with access to a balcony had a significantly different microbial community composition (p = 0.005) in comparison to outdoor cats. The same accounted for the beta diversity of indoor (which includes indoor, indoor with access to a balcony and shelter) compared with outdoor cats (p = 0.004). Principal coordinate analysis plots were constructed using the unweighted UniFrac metric to evaluate similarities in microbial communities defined as clustering by visual assessment. A large degree of variability was seen across all samples. Clustering in healthy cats was only observed for age groups and for different indoor/outdoor status (Fig 1). ANOSIM analysis based on unweighted and weighted UniFrac metrics did not detect a significant difference for the following factors: anesthesia (unweighted UniFrac distance p = 0.750, weighted UniFrac distance p = 0.794), same household (unweighted UniFrac distance p = 0.126, weighted UniFrac distance p = 0.2) or familial relationship (unweighted UniFrac distance p = 0.208, weighted UniFrac distance p = 0.209). Calculation of average distances showed no closer similarity between related cats and cats living in the same household when compared to unrelated cats and cats living in separate households.

**Fig 2:**
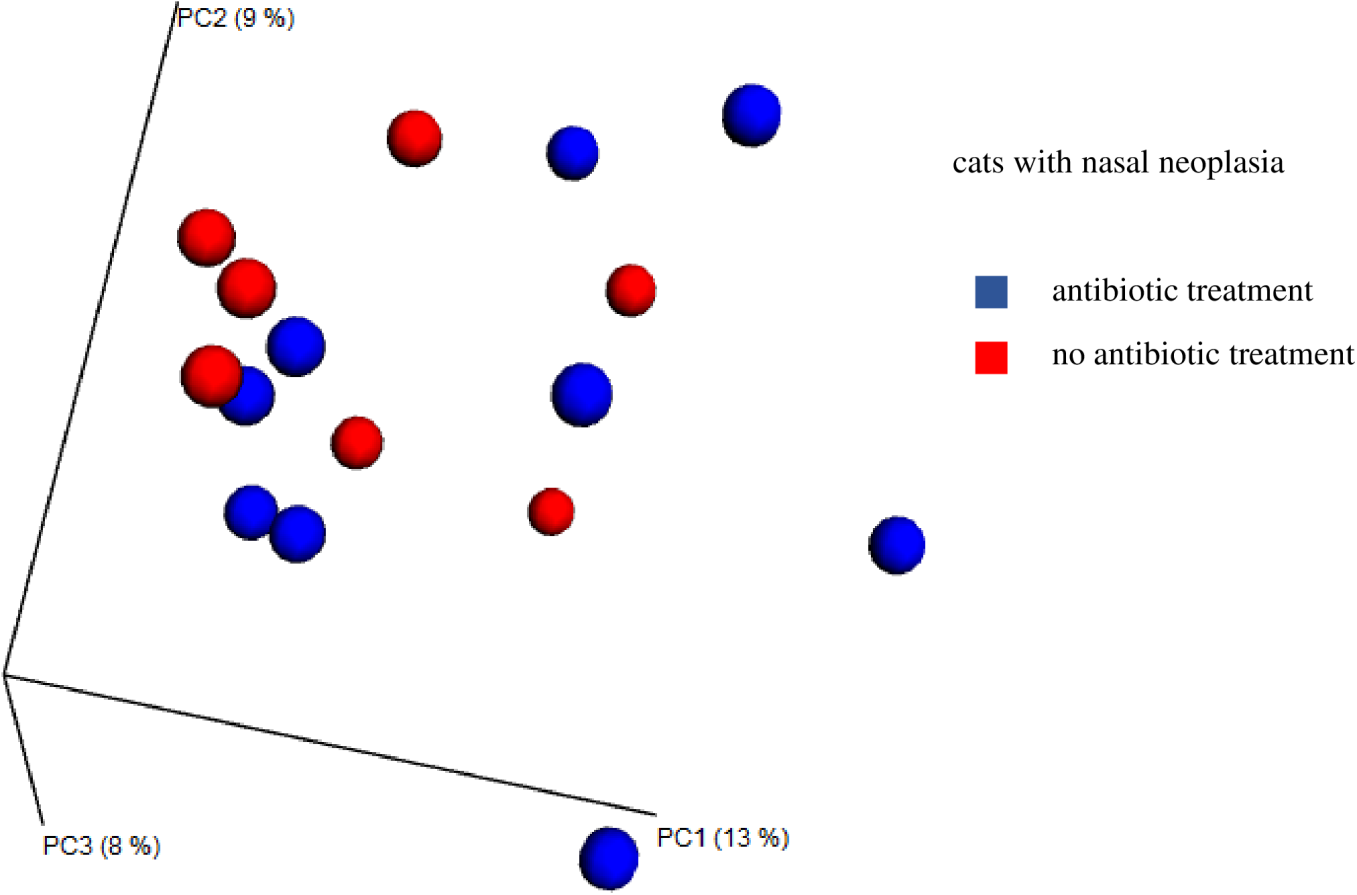
Principal coordinate analysis for antibiotic treatment of cats with nasal neoplasia. Principal coordinate analysis (PCoA) plots based on the unweighted UniFrac distance metric illustrating differences in microbial communities present in the nose of cats with nasal neoplasia. Every dot represents the bacterial community of one individual cat. A separation defined as clustering was not observed. PC1: first component along the x- and y-axes, PC2: second component, PC3: third component; blue dots: antibiotic treatment; red dots: no antibiotic treatment.

In cats with nasal neoplasia, unweighted UniFrac metric did not show a significant difference using ANOSIM analysis, when the microbiome of cats with nasal neoplasia that received antibiotics was compared to cats that did not have a history of antibiotic treatment (unweighted UniFrac distance p = 0.465, weighted UniFrac distance p = 0.159). Clustering based on similarities of bacterial molecular phylogenetic trees was not observed in comparisons of pretreated and untreated cats with nasal neoplasia (Fig 2).

**Fig 3:**
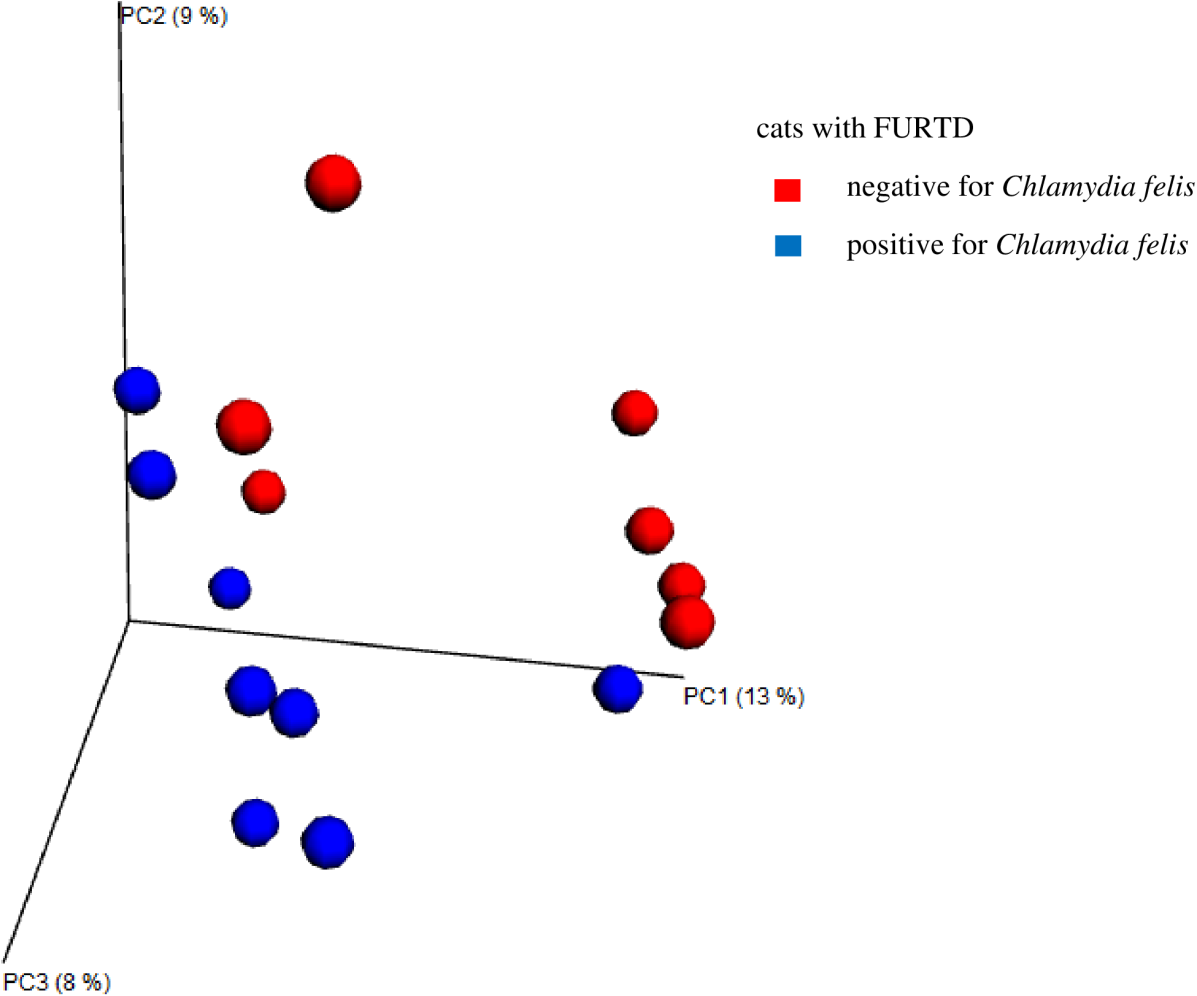
Principal coordinate analysis for Chlamydia felis infection in cats with FURTD. Principal coordinate analysis (PCoA) plots based on the unweighted UniFrac distance metric illustrating differences in microbial communities present in the nose of cats with FURTD. Every dot represents the bacterial community of one individual cat. A separation defined as clustering was observed in cats according to their status of being positive or negative for *Chlamydia felis*. PC1: first component along the x- and y-axes, PC2: second component, PC3: third component; blue dots: cats that tested positive for *Chlamydia felis*; red dots: cats that tested negative for *Chlamydia felis*.

**Fig 4:**
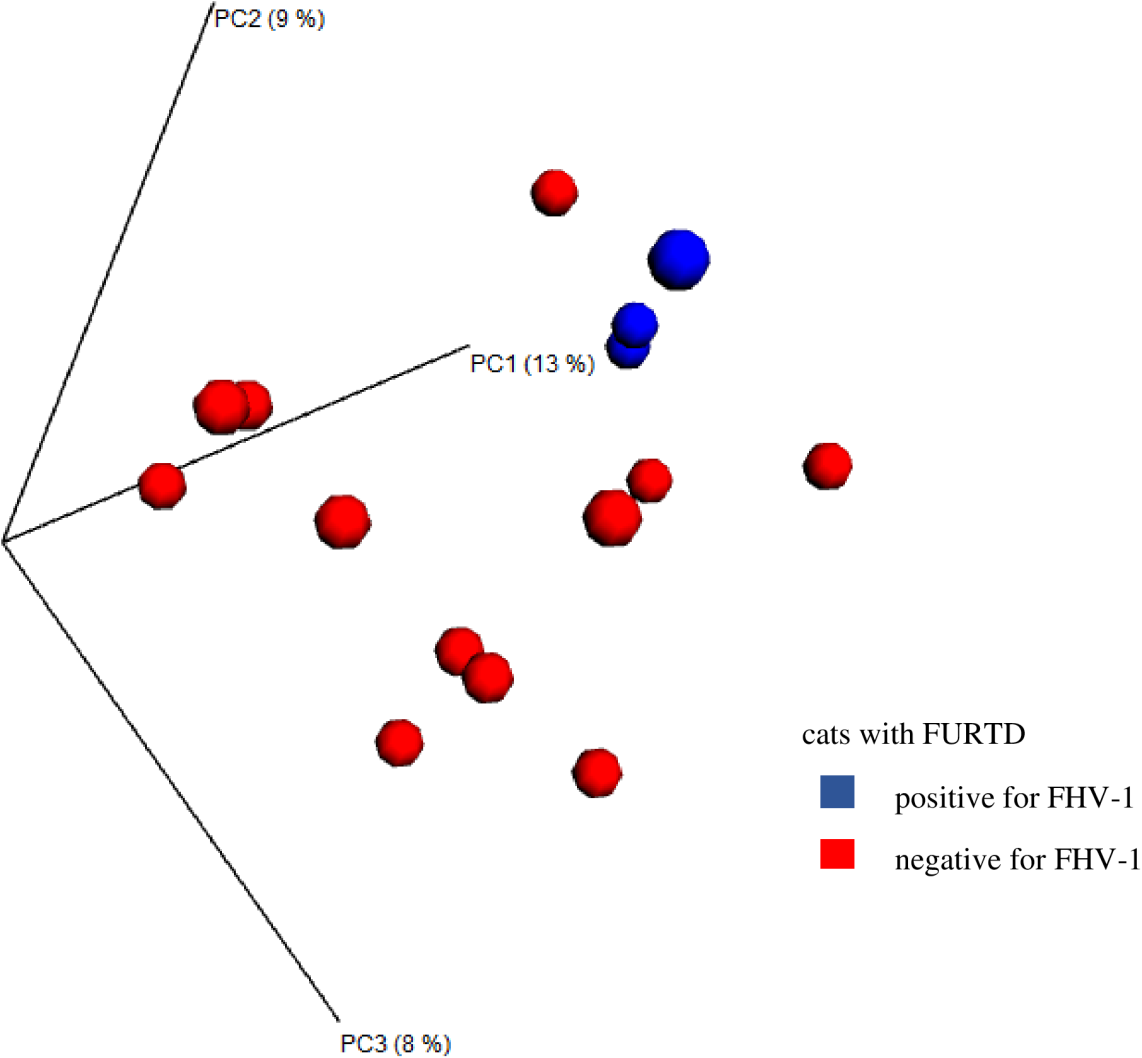
Principal coordinate analysis for FHV-1 infection in cats with FURTD. Principal coordinate analysis (PCoA) plots based on the unweighted UniFrac distance metric illustrating differences in microbial communities present in the nose of healthy cats that tested positive or negative for FHV-1. Every dot represents the bacterial community of one individual cat. A separation defined as clustering was observed. PC1: first component along the x- and y-axes, PC2: second component, PC3: third component; blue dots: cats that tested positive for FHV-1; red dots: cats that tested negative for FHV-1.

In cats with FURTD, beta diversity showed significant differences between cats with and without *C*. *felis* infection (Fig 3, p = 0.011) and in cats with and without FHV-1 infection (Fig 4, p = 0.033) for ANOSIM performed on the unweighted UniFrac distance. However, when the analysis was performed on the weighted UniFrac distance, no significant difference was observed (*C*. *felis* p = 0.088 and FHV-1 p = 0.092).

### Species richness and diversity

**Fig 5:**
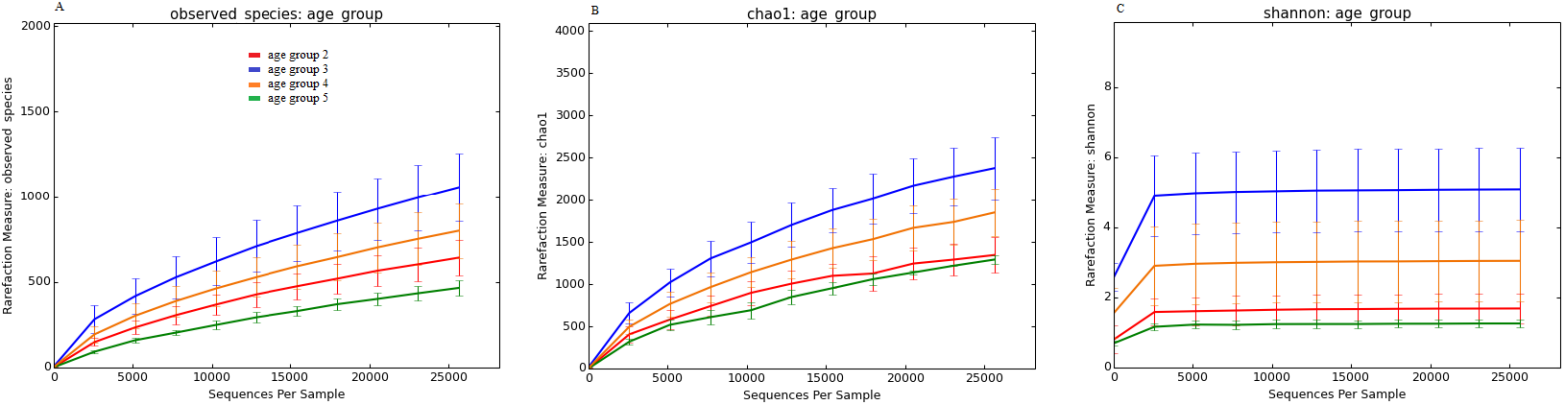
Rarefaction curves of 16S rRNA gene sequences from healthy cats of different age groups. Lines represent the mean of each age group, and error bars represent the standard deviations. The analysis was performed on a randomly selected subset of 25,640 sequences per sample. (A) Observed Species. (B) Chao1 index. (C) Shannon diversity index (age group 2: red line, age group 3: blue line, age group 4: orange line, age group 5: green line).

**Fig 6:**
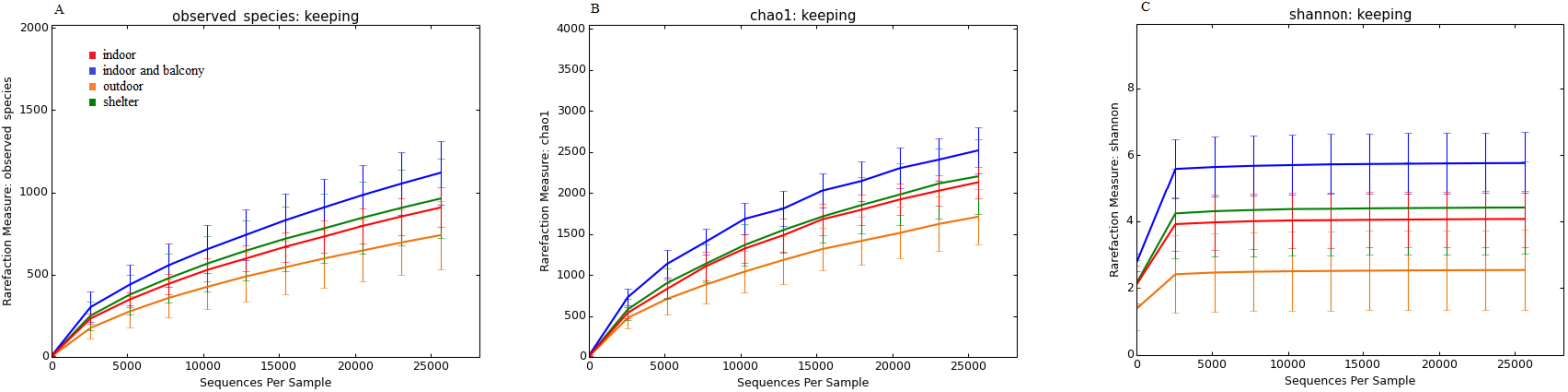
Rarefaction curves of 16S rRNA gene sequences obtained from healthy cats living in different environments. Lines represent the mean of each group, and error bars represent the standard deviations. (A) Observed Species. (B) Chao1 index. (C) Shannon diversity index (cats living indoor: red line, cats living indoor with access to a balcony: blue line, cats with access to the outside: yellow line, cats living in a shelter: green line).

Species richness (observed species and Chao1) and alpha-diversity (Shannon) revealed high individual variability between samples collected from healthy and diseased cats (S1 Table). As explained above, the current techniques for selecting OTUs are known not to concur with the original term “species”. In this context, the “observed species” metric is the number of unique OTUs found in the sample. Species richness was significantly higher in cats of age group 3 compared with age group 2 (Fig 5, p = 0.001) when all age groups of healthy cats were compared. When 25,640 sequences per sample were analyzed, the number of observed species ranged from 1,426 to 796 for age group 3 to the lowest number of 508 to 422 for age group 5. Cats kept indoors had a significantly higher number of observed species compared with cats with access to the outside (Fig 6, p = 0.042). The number of observed species ranged from 1,426 to 935 for indoor and balcony cats and was lowest for outdoor cats with 990 to 422.

As an estimator for species richness at a higher sequencing depth, the Chao 1 index also showed significantly higher levels for age group 3 when all age groups were compared (Fig 5, p<0.001). In comparisons of indoor/outdoor status, the Chao 1 index revealed that most indoor cats had a higher observed species number compared with outdoor cats (Fig 6, p = 0.011).

The Shannon diversity index, which takes into account the abundance and evenness of species, showed similar results concerning the number of observed species and Chao 1 index in different age groups, with age group 3 having the highest and age group 5 having the lowest diversity, and a significant difference between age group 2 and age group 3 when all age groups were compared (Fig 5, p<0.001). Similarly, samples from indoor cats were more diverse compared with outdoor cats based on the Shannon index (Fig 6, p = 0.003).

### Most common taxa colonizing the nose

**Fig 7:**
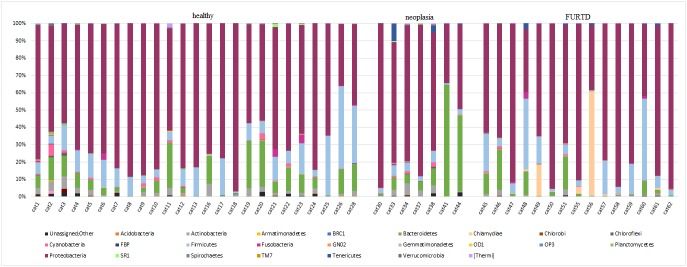
Composition of the nasal microbiome in healthy and diseased cats. Bacterial phyla in the nose of single cats enrolled in the study. Each bar chart represents one cat.

**Fig 8:**
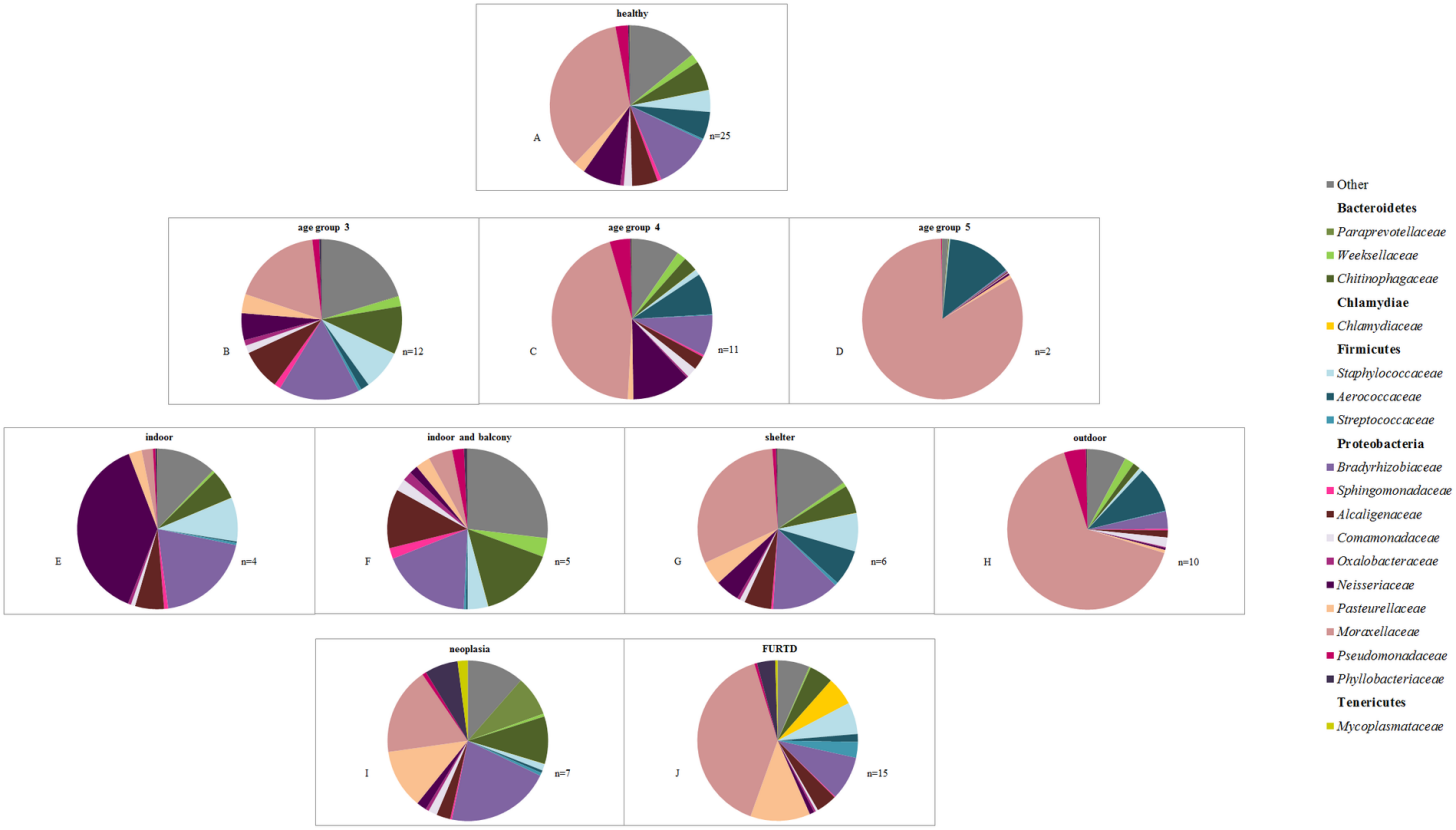
Bacterial phyla and families in the nose of cats. The average of the most common bacterial phyla and families identified in healthy cats according to their age group or environment and in cats with FURTD and nasal neoplasia. Healthy cats: A, B, C, D, E, F, G, H.

Twenty-four phyla were identified from all nasal samples. Proteobacteria were the predominant bacterial phylum in healthy and diseased cats. Proteobacteria, Bacteroidetes, and Firmicutes together represented, on average, more than 92% of the total bacteria sequenced in all three groups. At the finest taxonomic resolution possible, a total of 375 OTUs was found, but DNA could not always be resolved beyond the genus level (S2 Table). The composition of the nasal microbiome differed at an individual level (Fig 7). The predominant bacterial groups in the nose of healthy and diseased cats are displayed in Table 4 and Table 5 and Fig 8. S2 Table summarizes the proportions of bacteria by phyla, class, order, family, and genus in healthy cats, different age groups of healthy animals, healthy cats with different indoor/outdoor status and diseased cats.

**Table 4:** Relative proportions of the most abundant bacterial taxa identified by sequencing of the 16S rRNA gene.

	Healthy (n = 25)		Neoplasia (n = 7)		FURTD (n = 15)	
Taxa	mean %	SD	mean %	SD	mean %	SD
**Phylum**						
Bacteroidetes	9.3	8.3	19.5	24.5	5.7	7.7
Chlamydia	0.1	0.0	0.0	0.0	5.7	15.7
Firmicutes	13.0	11.2	3.8	2.0	13.1	14.3
Proteobacteria	71.4	14.5	68.8	21.2	72.7	21.1
**Class**						
Alphaproteobacteria	15.0	12.2	29.2	17.5	13.4	13.9
Betaproteobacteria	15.5	17.7	7.4	5.6	6.0	7.0
Gammaproteobacteria	40.7	31.0	32.1	26.3	52.9	32.0
**Order**						
Pseudomonadales	37.5	32.7	18.5	31.1	40.4	33.3
Rhizobiales	12.9	10.5	28.3	16.9	12.9	13.7
**Family**						
*Bradyrhizobiaceae*	11.4	10.2	21.0	18.8	8.8	12.7
*Moraxellaceae*	34.9	33.9	17.7	31.4	39.8	33.7
*Neisseriaceae*	7.8	17.3	2.0	3.3	0.9	0.8
**Genus**						
*Moraxella*	33.0	34.2	15.8	31.8	38.6	33.8
unclassified *Bradyrhizobiaceae*	11.3	10.1	20.6	18.4	8.8	12.6

**Table 5:** Taxa present at >1% mean relative abundance in one or more groups of cats enrolled in the study.

			Healthy (n = 25)		Neoplasia (n = 7)		FURTD (n = 15)	
Phylum	Family	Genus	mean %	SD %	mean %	SD %	mean %	SD %
Actinobacteria	*Corynebacteriaceae*	*Corynebacterium*	0.9	1.5	1.1	1.0	0.3	0.6
Bacteroidetes	*[Paraprevotellaceae]*	*[Prevotella]*	0.1	0.1	8.0	20.2	0.1	0.2
	*[Weeksellaceae]*	*Cloacibacterium*	1.6	2.9	0.5	0.6	0.2	0.6
	*Chitinophagaceae*		0.2	0.2	7.3	14.6	0.2	0.2
		*Sediminibacterium*	5.8	6.5	2.2	1.9	4.6	6.6
Chlamydiae	*Chlamydiaceae*	*Chlamydia*	0.0	0.0	0.0	0.0	5.7	15.7
Firmicutes	*Staphylococcaceae*	*Staphylococcus*	4.3	9.3	1.3	1.3	6.3	9.1
	*Aerococcaceae*	*Alloiococcus*	5.5	8.2	0.5	0.9	1.6	1.9
	*Streptococcaceae*	*Streptococcus*	0.3	0.3	0.6	0.7	2.8	10.1
Proteobacteria	*Bradyrhizobiaceae*		11.3	10.1	20.6	18.4	8.8	12.6
	*Phyllobacteriaceae*	*Phyllobacterium*	0.4	0.3	6.6	14.0	3.7	8.1
	*Alcaligenaceae*	*Achromobacter*	4.9	5.2	2.7	2.5	4.0	5.6
	*Comamonadaceae*	*Lampropedia*	0.0	0.0	1.0	2.7	0.0	0.0
	*Neisseriaceae*	*other*	1.7	3.1	1.2	2.6	0.5	0.5
	*Neisseriaceae*		5.4	16.7	0.6	0.6	0.3	0.3
	*Pasteurellaceae*	*Bibersteinia*	1.2	1.9	5.8	7.7	4.0	3.8
		*Haemophilus*	0.1	0.1	0.2	0.2	1.6	5.3
		*Pasteurella*	0.6	0.6	5.1	8.5	5.7	14.2
	*Moraxellaceae*	*Acinetobacter*	1.1	1.2	1.4	1.4	0.5	0.6
		*Moraxella*	33.0	34.2	15.8	31.8	38.6	33.8
	*Pseudomonadaceae*	*Pseudomonas*	2.3	7.4	0.7	0.6	0.5	0.7
	*Xanthomonadaceae*		0.1	0.1	1.2	2.9	0.1	0.1
Tenericutes	*Mycoplasmataceae*	*Mycoplasma*	0.0	0.0	2.0	3.8	0.5	0.8

Similar to the difference observed in beta diversity between healthy indoor and outdoor cats, the analysis of individual bacterial groups with LEfSe showed a different abundance of taxa when these groups were compared. Indoor cats had a significantly lower relative abundance of the genus *Moraxella*, while significantly higher levels were detected in the genera *Bradyrhizobium*, *Staphylococcus*, and *Pasteurella* higher.

**Fig 9:**
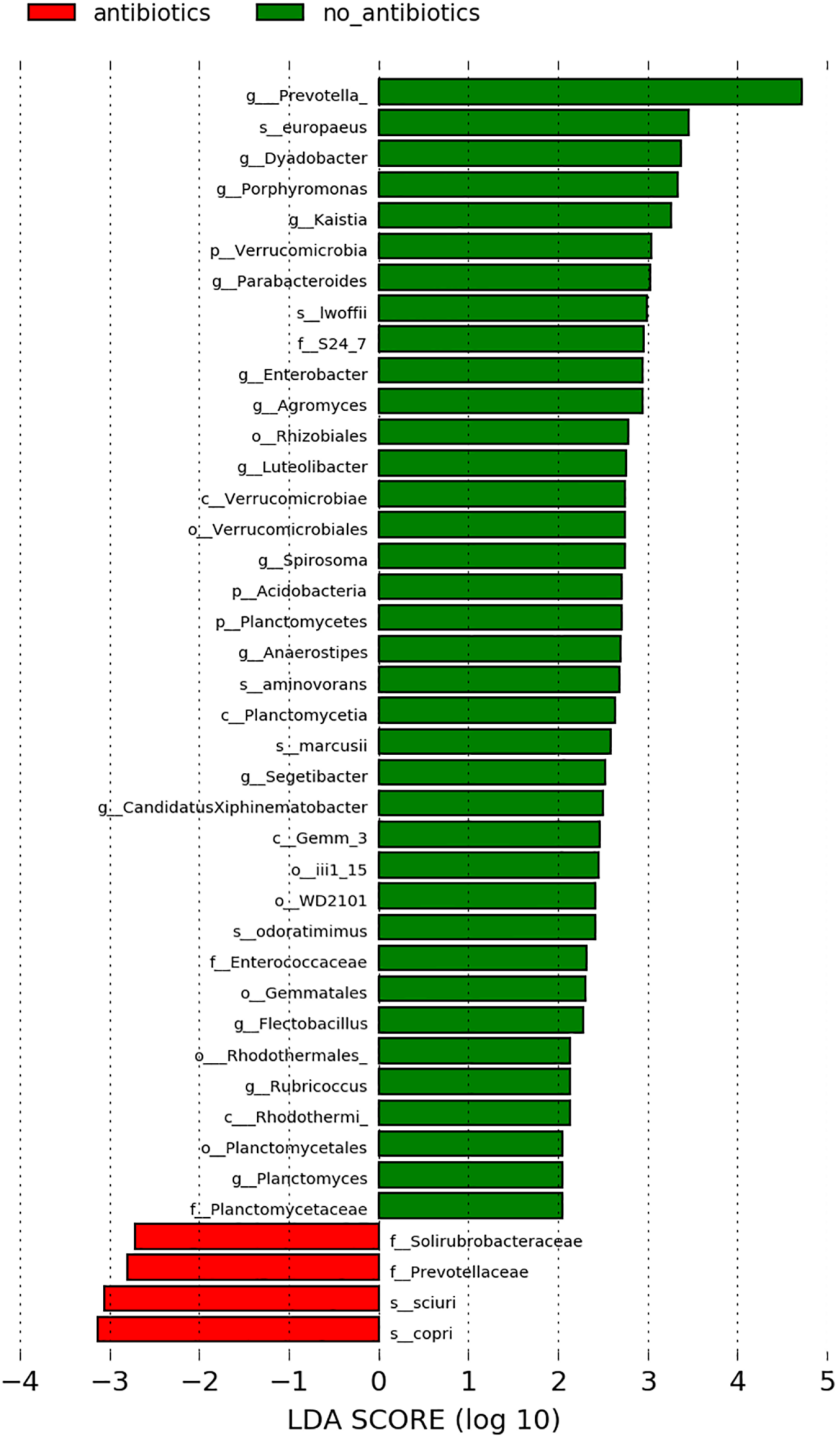
Differentially abundant bacterial groups shown by LDA scores based on LEfSe. Groups that were differentially abundant between cats with nasal neoplasia that received (red bars) or did not receive (green bars) antibiotic treatment before sampling. Taxonomic levels are represented as p_ (phylum), c_ (class, o_ (order), f_ (family), and g_ (genus).

Within the study group with nasal neoplasia, the bacterial taxa of patients with and without antibiotic treatment prior to sampling were compared. While there were no significant differences in alpha and beta diversity, several significantly different bacterial taxa could be established using LEfSe (Fig 9). While *Prevotella copri* and *Staphylococcus sciuri* were significantly more abundant in cats that had received antibiotics within the last 8 weeks, many taxa were decreased in cats with an antibiotic history.

## Discussion

The present study demonstrates that the nasal microbiome in cats is much more diverse than previously reported using culture-based methods [55–58]. Sequence data revealed a high individual variability among the samples collected from cats. Although additional samples from more cats are needed to arrive at further conclusions, the results suggest that the composition of the bacterial community is influenced by age and different environmental factors. In humans, a subject’s microbiome is personalized but includes dynamic changes throughout the first year of life characterized by a greater bacterial density and decreased diversity at a young age [3]. Similarly, the present study detected differences in the nasal microbial composition of healthy cats at different ages. Because of the strong influence of age on the nasal microbial composition, this parameter was not useful to compare healthy cats with diseased cats belonging to other age groups. In this case, differences in the microbiome could not only result from the underlying disease process but would also be affected by age. This phenomenon had to be kept in mind when interpreting the nasal microbial composition of cats with FURTD in the present study because FURTD is frequently detected in kittens younger than one year of age. In contrast, cats suffering from nasal neoplasia tend to be older. Because healthy cats had to be free from clinical signs of disease, few healthy old cats were eligible to enter the study. In the present study, the age of the healthy cats, cats with neoplasia, and cats with FURTD were significantly different. Therefore, a statistical comparison of the microbiome of cats with different disease statuses status was not performed.

In the present study, *Moraxella*, *Bradyrhizobiaceae*, *Sediminibacterium*, *Alloicoccus*, and *Neisseriaceae* were the most commonly detected bacteria in the nose of healthy cats. In cats with FURTD, *Moraxella*, *Bradyrhizobiaceae*, *Staphylococcus*, *Pasteurella*, *Chlamydia* and *Streptococcus* were the most frequently observed taxa. In previous studies using culture-based methods and PCR, the most commonly described bacteria in the nose of cats with FURTD included *Pasteurella*, *Streptococcus*, and *Staphylococcus* [57]. In addition to these bacteria, another study additionally detected *Mycoplasma* spp. as the most common bacterium in cats with FURTD [58]. Interestingly, in the present study, *Mycoplasma* was not one of the most abundant taxa in the nose of cats with FURTD concerning the relative abundance (mean 0.5%). *Chlamydia felis* (*C*. *felis*) is another known pathogen that is frequently detected in cats with FURTD and has been observed in the nose of cats with FURTD [59]. Notably in this study, *C*. *felis* was only observed in cats with FURTD (relative abundance 5.7%). *Staphylococcus* spp., *Pasteurella* spp., and *Streptococcus* spp. were commonly detected in cats with FURTD in this study, as previously described [57, 58]. The second most abundant taxa in healthy cats and in cats with FURTD detected in this study was *Bradyrhizobiaceae*, which has never been previously described as an inhabitant of the feline nose but has recently been detected in the feline oropharynx of healthy cats [42] and in the nasal cavity of healthy dogs [41]

The most commonly identified bacterial family in healthy cats and cats with FURTD was *Moraxellaceae*. In humans, *Moraxella catarrhalis* has been cultured from the hypopharynx of neonates as a risk factor for childhood asthma [60] and bronchiolitis or pneumonia [61]. During the first year of life, *Moraxella* spp. can be more frequently detected in children with acute respiratory infections than in healthy ones [62]. In cattle, *Moraxella bovis* and *Moraxella bovoculi* have been associated with bovine keratoconjunctivitis [63], but they could also be detected in the nasopharynx of asymptomatic cattle [64]. Studies investigating the feline oral microbiota in healthy cats [65] and in cats with and without periodontitis [66] identified *Moraxella* spp. as one of the core species of the oral cavity of healthy cats using next-generation sequencing. Similarly, the family *Moraxellaceae* also seems to represent the most abundant bacterial family in the feline nasal cavity. In a study including 59 cats with FURTD, *Moraxella* spp. was detected in aerobic cultures of 4 nasal and 21 pharyngeal swabs [58].

Members of the genus of unclassified *Bradyrhizobiaceae* were abundant in the nose of cats with nasal neoplasia and could also be detected in healthy cats. To the author’s knowledge, this genus has never been previously described in the feline nose. In humans, *Bradyrhizobium enterica* was found in colon biopsies of patients with cord colitis syndrome [67, 68], in the blood and lung of a patient with fatal pulmonary disease [69], and in blood samples of patients with poorly defined illness [70]. In animals, *Bradyrhizobium* could be detected in the gastrointestinal tract of the Amazonian catfish, (*Panaque nigrolineatus)* [71], yellow catfish (*Pelteobagrus fulvidraco*) [72], tropical caterpillars (Lepidoptera: Saturniidae) [73], and lagomorphs pikas (Ochotonidae) [74]. However, the role of *Bradyrhizobiaceae* in the feline upper respiratory tract and the organism’s potential role in the pathogenesis of nasal neoplasia in cats necessitate further studies.

The results of the present study indicate an abundance of *Pasteurella* spp., especially in diseased cats with nasal neoplasia or FURTD. In other studies, *Pasteurella* spp. were associated with feline gingivostomatitis [75] and have been isolated from wounds caused by cat bites [76].

Known bacterial pathogens that are frequently involved in FURTD, such as *C*. *felis* and *Mycoplasma* spp., were observed in diseased cats in the present study. *Mycoplasma* spp. have been detected by culture and PCR from nasal samples of healthy cats [36]. However, in most studies using culture or PCR for detection of *C*. *felis* and *Mycoplasma* spp. in healthy cats, conjunctiva and/or oropharyngeal region were sampled for pathogen detection [77–80]. Since the number of cats with FURTD was small in the present study, the roles of the pathogens FHV-1, FCV, and *C*. *felis* in the composition of the nasal microbiome during an acute phase of disease could not be assessed.

In the present study, the microbiome of healthy and diseased animals was not compared because a human [3] and a longitudinal study in pigs [43] showed significant dynamic changes in the nasal microbiome in early life as well as age-related differences in adults [35]. Furthermore, a longitudinal study examining the fecal microbiome of cats showed higher structural and functional diversity of the microbiome later in life compared with cats younger than 1 year [45].

It is often not clear which qualitative and quantitative changes in the microbiome are meaningful and whether these changes are associated with disease. Furthermore, distinguishing between cause and effect remains a challenge. It is still uncertain whether the microbiome in diseased animals is altered because of disease-related local or systemic immunosuppression or whether the altered microbiome is involved in the pathogenesis of certain diseases.

There are different interactions between the immune system and the microbiome. On the one hand, the immune system must learn to tolerate commensal bacteria, but on the other hand, it has to identify possible pathogens. These interactions of the host and its microbiome influence immune functions at all levels beginning at the initial innate defense response to acquired responses [81]. This phenomenon could be demonstrated in a study focusing on the upper airway mucosal lining fluid of the nasal cavity of human neonates. The presence of *Moraxella catarrhalis* as a potential airway pathogen was associated with an upregulated T helper cell (Th) type 1/Th2/Th17-type inflammatory response of the airway mucosa [82]. Another human study showed a stimulation of dendritic cells by *Moraxella*, leading to a three-to-five-fold increase in interleukin (IL)-23, IL-10, and IL-12p70 in comparison to stimulation by known respiratory commensal bacteria [83].As the microbiome influences the host immune response, one can speculate that a dysbiosis can cause disease, as has been shown for intestinal disease in dogs [84] or rhinosinusitis in a murine model [10]. It is also possible that a nasal disease process causes changes in the microbiome via different mechanisms, e.g., induction of mucosal inflammation toward a Th2-type response, interruption of immune defense [16], modification of epithelial barriers [85], mechanical obstruction and altered sinus functioning [86], including lower oxygen circulation in the upper airways caused by mucus [87], and swelling or blood vessel anomalies [88], as well as through the effects of medications [89]. Being able to describe the nasal microbiome in healthy animals and to identify possible changes that occur in the microbiome in disease could represent a first step in investigations of the role of the microbiome in the pathogenesis of diseases. As such, it could be a new way to investigate new diagnostic and therapeutic modalities.

The nasal microbial composition of cats with nasal neoplasia did not seem to be influenced by pretreatment with antibiotics according to the alpha and beta diversity. The only findings were differences in the abundance of taxa between both groups by LefSe analysis in patients who did not receive antibiotics, who presented a higher abundance of several taxa. These findings do not reflect the results of human studies indicating significant microbial changes caused by antibiotic usage [46–48]. However, the heterogeneous pretreatment and different types of nasal neoplasia within the population of cats with nasal tumors make it difficult to define a core microbiome of cats with nasal neoplasia in the present study, therefore potentially explaining the lack of antibiotic influence.

There are several limitations of this study. As mentioned previously, the groups were not age-matched, and therefore statistical comparisons of the nasal microbial composition between different disease and age groups were not useful. Another limiting factor of the study was the small number of cats included. Since the number of cats enrolled in each group was small, and significant variability was observed between individuals, a larger cohort of healthy and diseased cats should be evaluated to define the feline nasal microbiome and its role in health and disease.

## Conclusions

In conclusion, the present study revealed a large number of currently uncultivable bacteria, demonstrating that the nose of cats is inhabited by richer and more diverse microbial communities than has been previously described using culture-based methods. Furthermore, age and environmental factors seemed to influence the nasal microbial composition. Researchers are only just beginning to understand the complex interactions between the host and bacterial microbiota and the impact of disrupting this fragile homeostasis in disease states. The results of the present study represent a first step in the description of the nasal microbiome in healthy and diseased cats and the identification of intrinsic and extrinsic factors that influence the microbial composition. Future research in this field might help to develop new diagnostic tools to easily identify nasal microbial changes, relate them to certain disease processes, and help clinicians in the decision process of antibiotic selection for individual patients.

## Supporting information

S1 Table Alpha diversity. Alpha diversity measures at 25,640 sequences in healthy cats depending on age group and indoor/outdoor status and in diseased cats.
(XLSX)

S2 Table Relative percentages of bacterial groups. Relative percentages of bacterial groups in healthy and diseased cats at the various phylogenetic levels (phylum, class, order, family, genus) based on sequencing of the 16S rRNA gene.
(XLSX)
